# Optimising the Application of Multiple-Capture Traps for Invasive Species Management Using Spatial Simulation

**DOI:** 10.1371/journal.pone.0120373

**Published:** 2015-03-17

**Authors:** Bruce Warburton, Andrew M. Gormley

**Affiliations:** Landcare Research—Manaaki Whenua, PO Box 40, Lincoln, 7640, New Zealand; College of Agricultural Sciences, UNITED STATES

## Abstract

Internationally, invasive vertebrate species pose a significant threat to biodiversity, agricultural production and human health. To manage these species a wide range of tools, including traps, are used. In New Zealand, brushtail possums (*Trichosurus vulpecula*), stoats (*Mustela ermine*), and ship rats (*Rattus rattus*) are invasive and there is an ongoing demand for cost-effective non-toxic methods for controlling these pests. Recently, traps with multiple-capture capability have been developed which, because they do not require regular operator-checking, are purported to be more cost-effective than traditional single-capture traps. However, when pest populations are being maintained at low densities (as is typical of orchestrated pest management programmes) it remains uncertain if it is more cost-effective to use fewer multiple-capture traps or more single-capture traps. To address this uncertainty, we used an individual-based spatially explicit modelling approach to determine the likely maximum animal-captures per trap, given stated pest densities and defined times traps are left between checks. In the simulation, single- or multiple-capture traps were spaced according to best practice pest-control guidelines. For possums with maintenance densities set at the lowest level (i.e. 0.5/ha), 98% of all simulated possums were captured with only a single capacity trap set at each site. When possum density was increased to moderate levels of 3/ha, having a capacity of three captures per trap caught 97% of all simulated possums. Results were similar for stoats, although only two potential captures per site were sufficient to capture 99% of simulated stoats. For rats, which were simulated at their typically higher densities, even a six-capture capacity per trap site only resulted in 80% kill. Depending on target species, prevailing density and extent of immigration, the most cost-effective strategy for pest control in New Zealand might be to deploy several single-capture traps rather than investing in fewer, but more expense, multiple-capture traps.

## Introduction

Internationally, invasive vertebrate species pose a significant threat to biodiversity, agricultural production, infrastructure, and animal and human health [1], and a wide range of tools, including traps, are used to mitigate their negative impacts. In New Zealand, brushtail possums (*Trichosurus vulpecula*), stoats (*Mustela erminea*), and ship rats (*Rattus rattus*) among other species have become significant invasive species, and have had (and continue to have) significant impacts on indigenous plants and animals [2]. All three species are ubiquitous in New Zealand’s forests, and as agile climbers they put both ground and canopy nesting birds at risk from predation. Stoats are obligate carnivores and have relatively large home ranges (30–350 ha[3]). Possums are primarily folivores, but occasionally eat meat from carcasses and opportunistically predate eggs and nestlings. Possum home ranges in forest habitats are in the order of 2–5 ha[4]. Ship rats are omnivores and have home ranges in the order of 0.5 to 1 ha[5]. Control of these pests is carried out using aerial application of poison [6,7] and ground-based applications of poison and traps [8]. Recently, there has been an increasing demand for cost-effective ground-based control methods because of public opposition to the aerial application of 1080 poison [9], and the increased number of community groups involved with pest control programmes [10]. Although there is a range of poisons and traps available for ground-control, traps are often considered expensive to use because each trap can only capture a single animal at a time and requires frequent checking to clear and re-set. Multiple-capture traps have recently been developed to address the cost issue (e.g. A12 possum and A24 rat + stoat) and are now commercially available (http://goodnature.co.nz/). Because current and future multiple-capture traps are, or are likely to be, more expensive ($US125–140) than single capture traps ($US7-$25 depending on target species), it is important to understand the relative cost efficiencies of the different trap types in various situations. That is, knowing how many individual captures a trap might have at a single site over a fixed time period will enable prediction of how many potential captures a multiple-capture trap might require to obtain the same result, or alternatively if single capture traps are used, how many need to be set at a single site.

Effective management of possums, stoats and rats in New Zealand usually involves an initial knockdown of the target population to below some threshold density level at which that population no longer causes unacceptable impacts [11], followed by maintenance of the population at or below this density [12,13]. The initial knockdown phase can be achieved cost-effectively with a number of currently available poisons or traps, but the maintenance phase becomes expensive because of the continuing control effort that needs to be applied even when the number of animals captured is low. Consequently, if checking frequency can be reduced then there are potentially significant cost savings. However, in the maintenance phase of control when infrequent checking of traps would be beneficial, there are few residual animals to be caught at any one trap site. Spending scarce operational funds on traps that have the capacity to capture 10–20 individuals might not be justified if only a few individuals will be caught at any one trap site between the necessary trap checking time.

Our aim in this study was therefore to determine, for a given density of target pest animals and checking period, what would be the maximum number of captures that any one trap might achieve. Such information would enable pest controllers to make choices between the need for expensive multiple-capture traps and the possible alternative of using multiple cheaper single-capture traps. Additionally, such information will enable the developers of multiple-capture traps to consider what an optimal number of captures the traps should be designed to deliver, in order to avoid designing redundant capture capacity that might make the trap more expensive than required.

Field-based comparisons of trap efficiency at low animal densities are expensive because a large number of trap-nights are required to capture sufficient animals to obtain acceptable statistical power, so we chose to initially model the different trap options using an individual-based spatially explicit modelling approach [14].

## Methods

### Model parameters and simulations

An individual-based simulation model, based on the spatially explicit possum model of Ramsey and Efford [14], was developed in R version 2.15.2 [15]. It assumes animals occupy circular home ranges that are randomly distributed across the sample frame, but fixed for the duration of the trapping period. The probability (P) of an animal (i) being caught in a particular trap (j) on a given night declines with the distance *d*_*ij*_ between the centre of its home-range and the trap [16]. Thus:

$$P\;=\;g\left(0\right)e^{\left(-d_{ij}^{2}/2\sigma^{2}\right)}$$ Eq. 1

This relationship is a half-normal detection function, where g(0) is the probability of capture when the trap is located at the centre of the home range, and σ is a measure of home-range size (2.45σ equals the radius within which the animal is likely to be found 95% of its time [17]).

Traps for possums, stoats and rats were located in a grid across a simulated area with the spacing between trap-lines and between traps along lines following current best practice (Table 1). At the start of each simulation individual animals were randomly located within the trap-grid area at a specified mean density per ha based on typical maintenance densities for each of the target species (Table 1). A pre-specified buffer area was also placed around each simulated control area to enable animals at the edge of the control area to still have full circular home ranges (Table 1). The distance between each trap and animal was calculated and the subsequent daily probability of capture between each trap and animal determined using Eq. 1. Because the simulation modelled capture probabilities rather than actually killing, from here on the term capture is used as opposed to kill. The model ran for 30 simulated nights of trapping, with captured animals ‘removed’ from the population each night. The number of animals captured per trap was recorded and traps that reached the maximum specified trap catch for each trap type were removed from service for the remainder of the 30-night trapping period. That is, traps were not checked each night. For single-capture traps, traps were ‘removed’ after capture of a single animal, whereas for multiple-capture traps, these traps remained in service until their capacity was reached (e.g. 6 or 12 captures), or the end of the trapping period occurred.

**Table 1:** Parameter values used in the modelled simulations for possums, ship rats, and stoats.

Parameter	Possum	Stoat	Ship-rat
g_0_	0.05	0.045	0.03
σ	63	641	32
Density range (animals/ha)	0.5–3.0	0.02–0.12	1.0–11.0
Between trap spacing (m)	50	200	25
Between line spacing (m)	100	800	100
Buffer (m)	100	300	100
Immigration rate	NA	NA	Density/30
Trap area (ha) (excluding buffer)	1007	1040	1014
Trap area (ha) (including buffer)	1140	1472	1148
Trap sites	2106	84	4200

Trap-setting parameter values based around current best practice guidelines for vertebrate pest control in New Zealand [26].

Values for the parameters of the half-normal detection function (g_0_ and σ) (Table 1) were, for possums, taken from [16], stoats [18] and rats [19].

The size of the simulated trapped area varied between species (Table 1): for possums and stoats the area was set to c. 1000ha, but for rats, which had simulated densities up to 11/ha, the area was reduced to about 500ha to make the simulations computationally tractable. The initial modelling used a closed area (i.e. no immigration), but this was then modified for ship rats only to assess the effect of immigration on model predictions. Thus for rats, the modelling was repeated assuming daily immigration equivalent to a population equal to the initial density being introduced over a period of 30 days (i.e. daily immigrants = density/30). To simulate the likely gradient of immigrants from the perimeter to the centre of the simulated area, we modelled three equidistant steps with 60%, 30%, and 10% of immigrants having home ranges in the outer, middle, and inner areas respectively.

### Simulations runs and cost estimations

Each simulation was run for 30 days using 100 replicates to generate the number of traps that caught 0,1,2…N captures. To compare the costs of multiple- versus single-capture traps we used the price per trap as well an estimate of time for setting up and checking the traps (Table 2). The times used were means of times obtained from six agency field control staff who were familiar with a range of trap models. We used a daily contract rate of $NZ300.

**Table 2:** Indicative costs for each trap type simulated and times for establishing and checking them.

Trap model	Price ($US)	Set-up time (min)	Checking/reset time (min)
Sentinel^1^	$24.00	5	2
Goodnature A12 & A24^2^	$140.00	5	3
Victor Easy Set Snap-back trap^3^	$6.30	4	2
DOC 150 plus wooden tunnel^4^	$38.00	3	3

^1^ http://www.pestcontrolresearch.com

^2^ http://goodnature.co.nz/

^3^ http://www.nopests.co.nz

^4^ http://www.predatortraps.com

### Data accessibility

Output data from the modelling simulations, as well as the R code used to generate the model, are publicly accessible at: http://dx.doi.org/10.7931/J28G8HMC (which is a site hosted by the parent organisation, Landcare Research Ltd.) or alternatively can be accessed via doi: 10.7931/J28G8HMC.

## Results

### Possums

**Fig 1:**
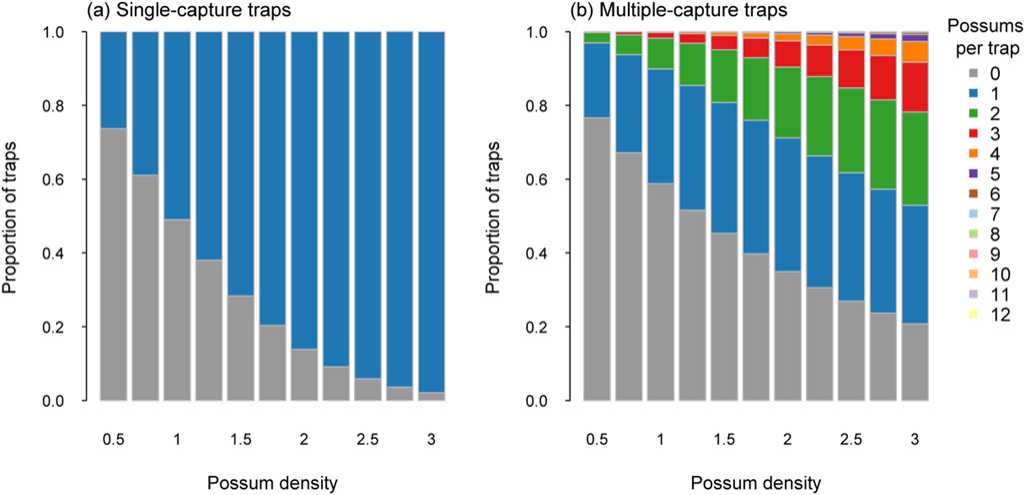
Proportion of traps catching a specified number of possums for single-capture traps (a) and multiple-capture traps (capacity 12; b) for possum densities from 0.5 to 3.0 possums per ha.

The trap layout for modelling possum captures resulted in 2106 trap sites being allocated across the 1000 ha simulated area (Table 1). At the lowest density of 0.5 possums/ha (i.e. 570 animals present in the simulated area), 26% of single-capture traps captured a possum (Fig. 1a). For multiple-capture traps with a capacity of two captures, 21% captured one possum and only 3% captured two possums. When the capacity was increased to 12 captures per trap, only 0.3% captured 3 or more possums, with the proportion of such traps capturing one or two possums being similar to the multiple-capture trap with a capacity of only two possums (Fig. 1b).

When possum densities were higher (i.e. 3 possums/ha), there were 3420 animals available to be captured and captured. At these higher densities, 98% of single-capture traps captured a possum (Fig. 1a). Multiple-capture traps with a capacity of two resulted in 62% of traps capturing two possums and 25% capturing one possum. For the multiple-capture traps with the potential of 12 captures per trap, 8.5% of the traps captured four or more possums, with most capturing either one (32%), two (25%) or three (13.5%) possums (Fig. 1b). Less than 1% of traps captured six or more possums (Fig. 1b).

**Fig 2:**
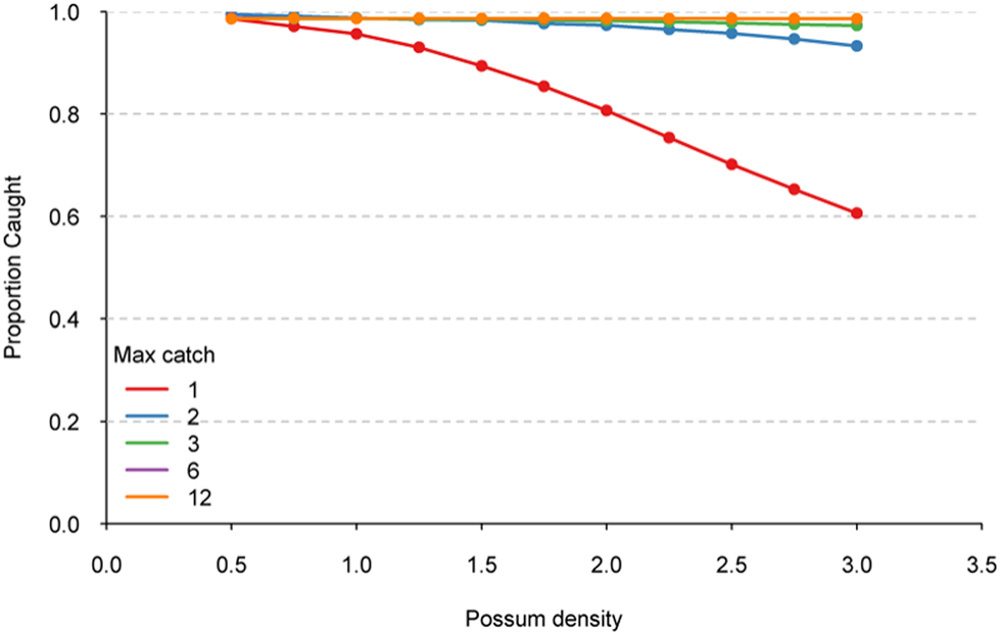
The proportion of the simulated possum population captured by traps with capture capacity ranging from 1, 2, 3, 6, and 12 captures at different possum densities.

At low possum densities, increasing the trap capture capacity had little effect on the proportion of animals captured (Fig. 2), with >98% of possums captured when possum densities were 0.5 possums/ha, regardless of the trap capacity. Efficacy of single-capture traps decreased with increasing possum density, mainly because the number of possums was greater than the number of traps available. When trap potential capture capacity was increased to at least three possums, greater than a 97% capture rate was achieved for the entire range of possum densities simulated (Fig. 2). Increasing trap capacity to 12 resulted in only small gains in capture percentage (e.g. 98.6% for 12-capture traps at 3 possums/ha compared to 97% for 3-capture traps).

### Stoats

**Fig 3:**
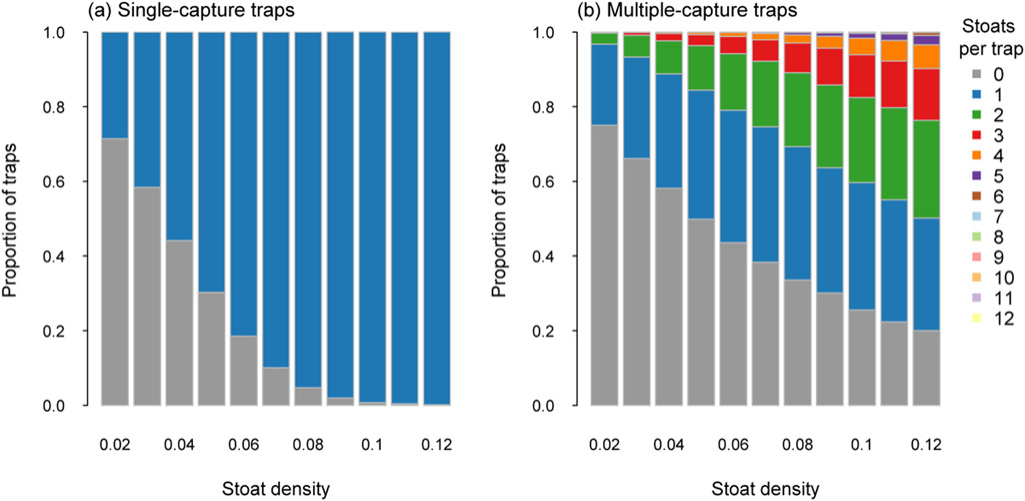
Proportion of traps catching a specified number of stoats for single-capture traps (a) and multiple-capture traps (capacity 12; b) for stoat densities from 0.02 to 0.12 stoats per ha.

For modelling stoat trapping, the layout of traps resulted in 84 traps being distributed across the simulated area. At the lowest density simulated of 0.02 stoats/ha (i.e. 29 animals), 29% of single capture traps captured a stoat (Fig. 3a). For multiple-capture traps with a capacity of two captures, 22% caught one stoat and 3% caught two stoats. When the capacity was increased to 12 captures per trap, only 0.3% of the traps caught 3 or more stoats, while 22% and 3% of the traps captured one or two stoats, respectively.

When stoat densities were higher (0.12 stoats/ha), there were 177 animals available to be captured. At these higher densities, 99.9% of single-capture traps captured a stoat indicating almost total trap saturation. Increasing the capacity to two captures resulted in 74% of traps capturing two stoats and18% capturing one. For the multiple-capture traps with 12 potential captures per trap, 10% of such traps caught four or more stoats, with most catching either one (30%), two (26%) or three (14%) stoats (Fig. 3b).

**Fig 4:**
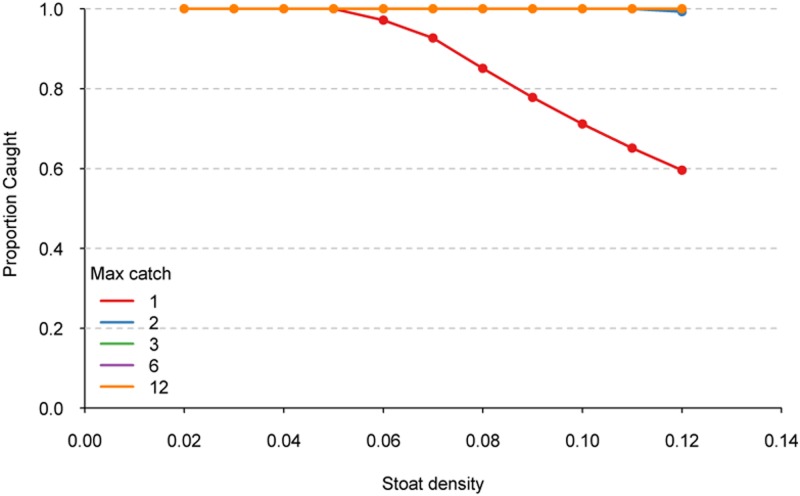
The proportion of the simulated stoat population captured by traps with capture capacity ranging from 1, 2, 3, 6, and 12 captures at different stoat densities.

At low simulated stoat densities, increasing the trap capacity had little effect on the proportion of animals captured (Fig. 4), with c. 100% of animals captured when densities were 0.02 stoats/ha, regardless of the trap capacity. Efficacy of single capture traps decreased greatly with increasing stoat density, with only 60% of the population caught in single-capture traps at the highest stoat densities. When trap capacity was increased to at least two stoats, greater than 99% capture rates were achieved for the entire range of stoat densities simulated (Fig. 4). Increasing trap capacity to 12 captures only resulted in small gains in capture percentage (c.100% for 12-capture traps at 0.12 stoats/ha compared to 99% for 2-capture traps).

### Rats

For modelling the trapping of rats, the trap layout resulted in 4200 traps being allocated across the simulated area.

#### With no immigration

Rats were modelled with the highest densities of the three species simulated, ranging from 1 to 11 rats/ha, (corresponding to 1148 and 12,628 rats respectively). At the lowest density simulated (1 rat/ha), 22% of single-capture traps captured a rat. For multiple-capture traps with a capacity of two, 80% of traps caught no rats, 18% caught one rat and 2% caught two. When the capacity was increased to 12 captures per trap, only 0.2% caught 2 or more rats with 79%, 18% and 2% of traps capturing zero, one or two rats respectively.

**Fig 5:**
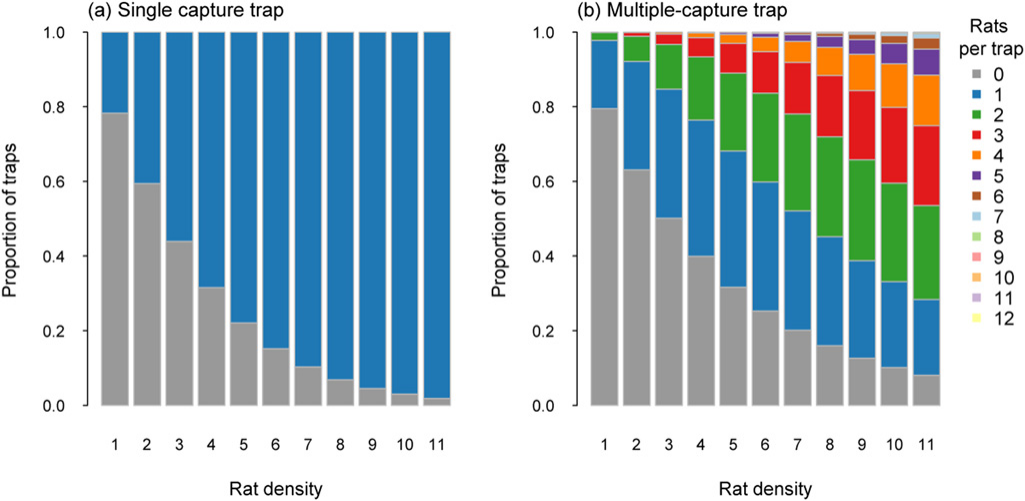
Proportion of traps catching a specified number of rats for single-capture traps (a) and multiple-capture traps (capacity 12; b) for rat densities from 1 to 11 per ha, with no immigration.

At the highest simulated rat densities (11 rats/ha), 98.2% of single-capture traps captured a rat indicating almost total trap saturation. Increasing the potential capture capacity per trap to two captures resulted in 82% of traps capturing two rats and 13% capturing one. For the multiple-capture traps with 12 potential captures per trap, 20% captured 1 rat, 25% 2rats, 22% 3 rats and 25% caught four or more rats (Fig. 5b). At the highest densities, most traps (95%) had captures of five or less rats (Fig. 5b). At maintenance densities of one or two rats/ha, most traps only caught one or two rats (Fig. 5b).

**Fig 6:**
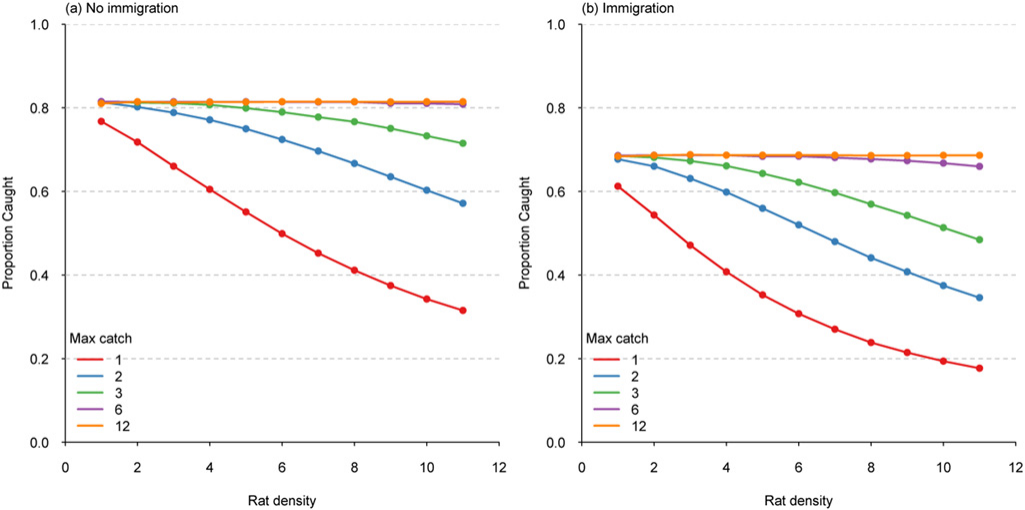
The proportion of the simulated rat population captured by traps with capture capacity ranging from 1, 2, 3, 6, and 12 captures at different rat densities assuming no immigration (a) and with immigration (b).

At low simulated rat densities, increasing the trap capture capacity had little effect on the proportion of animals captured (Fig. 6), with 77% of rats captured over 30 days using single capture traps, increasing to 81% with multi-capture traps. Efficacy of single-capture traps decreased greatly with increasing rat density, trapping only 32% of the population when at the highest rat densities. When trap capacity was increased to at least three rats per capture, greater than a 71% capture rate was achieved for the entire range of rat densities considered, and greater than 80% when trap-capacity was increased to at least six rats (Fig. 6). At the highest rat densities, increasing trap capacity to 12 resulted in small gains in capture percentage (81% for 12-capture traps compared to 72% for 3-capture traps).

#### With immigration

When immigration was included as a confounding variable, a higher proportion of single capture traps captured a rat than without immigration. At the lowest density simulated (1 rat/ha), 32% of single-capture traps captured a rat. For multiple-capture traps with a capture capacity of two, 25% caught one rat and 5% caught two. When the capacity was increased to 12 potential captures per trap, 0.5% caught 3 or more rats with 25% and 4.5% of traps capturing one or two rats, respectively.

**Fig 7:**
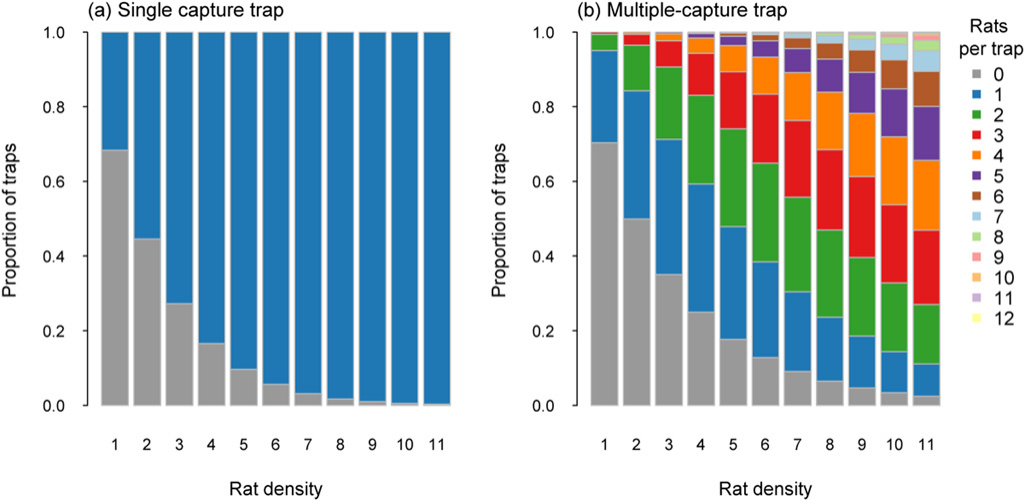
Proportion of traps catching a specified number of rats for single-capture traps (a) and multiple-capture traps (capacity 12; b) for rat densities from 1 to 11 per ha, with immigration.

The higher proportion of traps with captures was also apparent at higher densities of rats. At the highest simulated rat densities (11 rats/ha), there were 12,628 animals available to be captured. At these higher densities, 99.7% of single-capture traps captured a rat indicating almost total trap saturation. Increasing the capture capacity to two captures per trap resulted in 96% of traps capturing two rats and 3.5% capturing one. For the multiple-capture traps with 12 captures per trap, 9% captured 1 rat, 16% 2 rats, 20% 3 rats and 52% caught four or more rats (Fig. 7b).

At the highest rat densities, 80% of multiple capture traps had captures of five rats or fewer (Fig. 7b). At maintenance densities of one or two rats/ha, most traps only caught one or two rats (Fig. 7b). At low simulated rat densities, increasing the trap capture capacity above two rats had little effect on the proportion of animals captured (Fig. 6), with 61% of rats captured over 30 days with single capture traps, increasing to 68% with multiple-capture traps. The efficacy of single-capture traps decreased greatly with increasing rat density, since only 18% of the rat population was caught using single-capture traps at the highest rat densities. Increasing the trap capacity to three resulted in at least a 50% capture rate for the entire range of rat densities considered, and to greater than 65% capture rate when trap-capacity was increased to at least six (Fig. 6). At the highest rat densities, increasing trap capacity to 12 resulted in small gains in capture percentage (69% for 12-capture traps compared to 66% for 6-capture traps, at 11 rats/ha). At the highest rat densities the capture rate using 6-capture traps was substantially higher than for 3-capture traps (Fig. 6b).

### Cost

**Fig 8:**
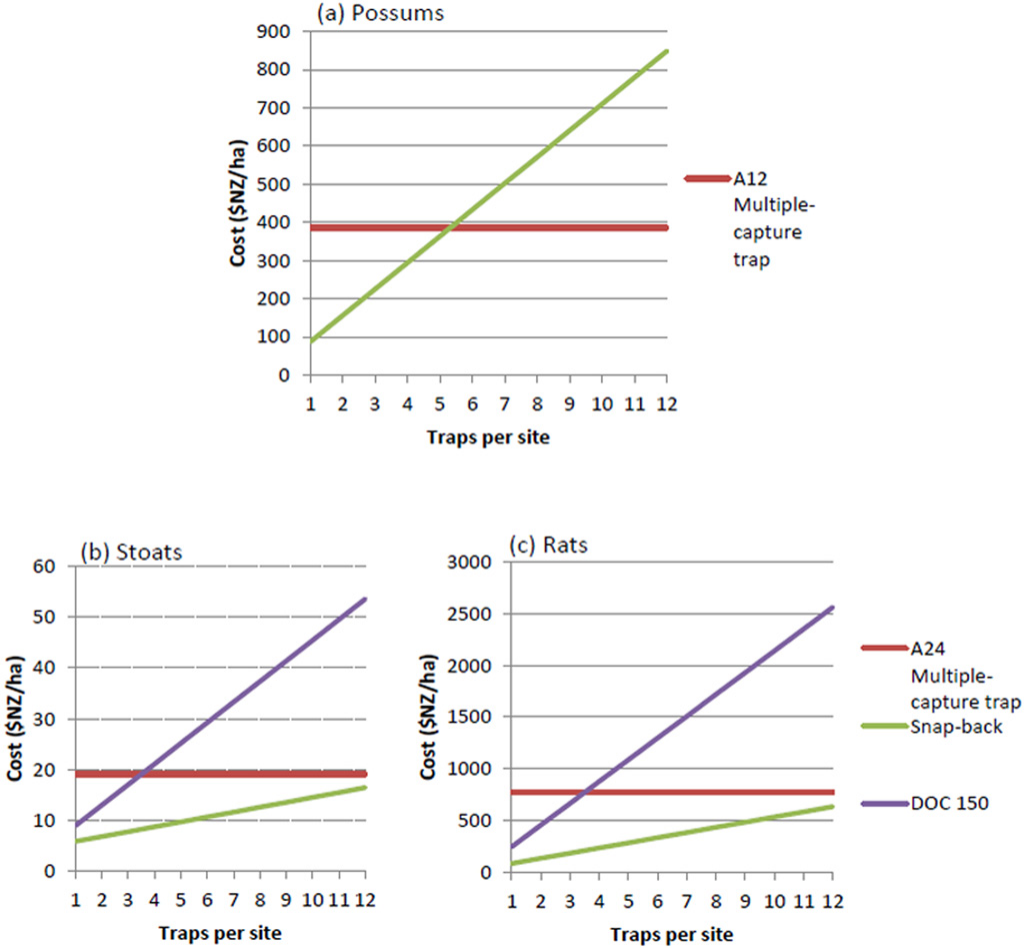
Costs per hectare of using multiple-capture traps and a varying number of single-capture traps for (a) possums, (b) stoats, and (c) rats.

The choice of whether to invest in relatively expensive multiple-capture traps or several cheaper single-capture traps depends on the price of the traps and, if using single-capture traps, how many are placed at each site. For possums we compared the Sentinel (a single-capture trap) and the Goodnature A12 (a multiple-capture trap). Fig. 8a shows that setting five Sentinel traps at each site has a similar cost as setting a single Goodnature A12 trap (capacity to kill 12 possums) at each site, assuming each trap type has the same capture efficiency. For maintenance control of possums, when densities will be less than 2 possums/ha, setting three or at most four Sentinel traps per site will be more cost-effective than setting the more expensive A12 multiple-capture traps.

For stoats we compared the Goodnature A24 (capacity to kill 24 stoats or rats), and the DOC 150, and the Victor snap-back trap (both single-capture traps). The simulations showed that only two traps need to be used at a site to catch 99% of stoats even at the highest densities simulated (Fig. 4), and this option would be cheaper than using A24 traps (Fig. 8b). Consequently, most stoat populations could be managed at a trap cost of about $US11/ha using DOC 150 traps rather than $US17/ha if using A24 traps (Fig. 8b). Similarly, if the much cheaper snap-back traps are used, three of these could be used per site at a cost of only $US6.50/ha (Fig. 8b).

For ship rats we compared the same traps as for stoats, but because of the higher densities of rats and the higher density of traps used, the costs per hectare were considerably higher than for trapping stoats (Fig. 8c). In the worst case (i.e. with immigration and at the highest rat densities of 11/ha) six traps at a site caught 66% of rats compared to 69% if twelve captures per site were available. Consequently, if using six captures per site the DOC 150 traps would be the most expensive option ($US1100/ha), followed by the A24 ($US650/ha), and then the snap-back traps ($US280/ha) (Fig. 8c).

## Discussion

The individual-based simulation models provided an efficient method for determining the distribution of the number of captures likely to be obtained at any one trap site and therefore the likely maximum numbers of captures expected. This information could then be used to assist in designing optimal trapping strategies or informing the design of multiple-capture traps. For possums, that are generally maintained at densities less than one per hectare as a result of active population control [20], and for stoats that occur only at very low densities anyway [21], the modelling suggests that having the capacity to capture a maximum of three animals per trap-site will capture more than 97% of both species. Even at the highest densities simulated for possums (i.e. 3/ha) and for stoats (i.e. 0.12/ha), the percentages of the population caught with three traps per site were 97% and 99%, respectively. If a maximum of three captures per site could achieve the operational goal then using three individual traps per site is also more cost-effective than the more expensive multiple-capture trap option. These results, however, are based on simulating a closed population and trapping for 30 days. However, leaving the traps open for a longer period would have very little, if any, further impact on the *in situ* population, but would place any potential immigrants at risk of capture. For possums and stoats, however, the number of immigrants over such a time-frame is likely to be low [22] although this would depend on adjoining habitat, source population density and season. Because traps need to be maintained, including ensuring any lure or bait in still fresh and attractive, we selected 30 days as a realistic period to leave traps active between checks. With the development of slow-release, long-life lures [23,24], checking periods could be extended, perhaps out to six months or more. If immigration was sufficiently high then having higher capture capacity per site might be justified, but might only be required around the periphery of the operational area where immigrants would be first exposed to them.

Rats, predominantly ship rats (*Rattus rattus*) occur in similar densities to possums, but because of their high rates of reproduction, population increase and reinvasion potential they are more difficult to maintain at reduced densities [25]. Consequently we simulated a wider range of rat densities and also included immigration as a confounding factor in the simulations. Although we used operational best practice guidelines for trap spacing and spacing between trap lines, the simulation results indicated that for rats the trapping intensity was insufficient to place all individuals in the population at risk. That is, even at the lowest rat density simulated (i.e. 1 rat/ha) the highest capture achieved was 81% with the multiple-capture traps. This suggests that either the sigma values used for rats in the simulation (i.e. 32m, Table 1) was too small, or operational trap density needs to be increased.

Simulated rat densities of 3/ha with no immigration, and using three potential captures per site, resulted in 81.1% of the rat population being caught, which was very similar to the 81.3% when trap sites had 12 potential captures. Even with immigration and trapping at densities of three rats per hectare, using three potential captures per site, 67.3% of rats were caught compared to 68.8% when using 12 potential captures. These results suggest there is no operational value in having trap sites with capture capacity above three if the rat density is at or below 3 rats/ha. At rat densities of 10/ha, the reduced proportion of the rat population captured using three potential captures per site (i.e. 51.3% of the population) compared to using 12 potential captures per site (68.6% of the population) would significantly affect the rate that the residual fast-breeding rat population could recover, and would therefore justify additional control effort. However, if rat densities were indeed high (10/ha and above) it would imply that either the control strategy being used had failed or, if an initial knockdown is required, that alternative control methods (e.g. poisons) should be used to achieve this.

In our simulations we had assumed that the capture probability (i.e. g(0)) for possible single- and multiple-capture traps was equivalent, even though in practise most traps differ in their capture efficiency [26,27]. In order for the simulations to accurately reflect the reality of commercially available traps, g(0) values would ideally need to be generated for each trap type. These values are not available for most traps or target species [17]. Differences in g(0) values might change the projected relative cost-effectiveness of multiple single-capture traps and single multiple-capture traps. However, the current simulations indicate that, unless the g(0) values differ markedly, for the purposes of maintaining low population densities the use of three or four single capture traps at a site would be more cost-effective than using expensive multiple-capture traps.

Making choices between multiple-capture and single-capture traps will depend on their component cost as well as their relative capture efficiency as discussed above. The current price of the multiple-capture traps are $US140 compared to $US18.90 to $US114, if three single-capture traps are required per site. This cost difference provides the users with savings they could potentially invest in controlling pests over a greater area. Using single-capture traps also provides the user with greater flexibility—for example, if the control area requires 100 trap sites, then when using multiple-capture traps 100 would be required. However, if using three single-capture traps at each site, 300 traps would be required, but as these do not have to be deployed in sets of three at each site they provide greater flexibility in locating traps throughout the control area and relocating some of them elsewhere if pest population densities are reduced to very low levels.

Multiple-capture traps such as the Goodnature A12 and A24 might provide a cost-effective option for perimeter protection during a pest control operation, by reducing the rate that immigrants establish in a control area and at which these can contribute to the acceleration of population recovery [28]. However, field trials would need to be carried out to determine how best to integrate multiple-capture traps into a trap-based control programme. Until this information is available, careful consideration should be given to which traps pest management agencies should invest in.

The simulations used here for determining optimal trap use could also be applied to optimising toxin delivery in static bait-stations, and for testing trapping and bait-station strategies for other pest species, including those with an environmental impact within and beyond New Zealand (e.g. feral cats). For example, if an acute toxin such as Feratox (potassium cyanide) is used in bait-stations [29], the simulation results show that for maintaining possum densities at low levels no more than three Feratox pellets would be required per station. Limiting the number of toxic pellets per station would enable users to reduce the amount of toxin applied to the environment and potentially reduce the cost of poison operations.
